# Correction: Opposing p53 and mTOR/AKT promote an in vivo switch from apoptosis to senescence upon telomere shortening in zebrafish

**DOI:** 10.7554/eLife.111193

**Published:** 2026-03-04

**Authors:** Mounir El Maï, Marta Marzullo, Inês Pimenta de Castro, Miguel Godinho Ferreira

**Keywords:** Zebrafish

El Maï M, Marzullo M, de Castro IP, Ferreira MG. 2020. Opposing p53 and mTOR/AKT promote an in vivo switch from apoptosis to senescence upon telomere shortening in zebrafish. *eLife* **9**:e54935. doi: 10.7554/eLife.54935.

Published 19 May 2020

We were alerted via a PubPeer post to inadvertent duplications of images in El Maï et al., 2020 (eLife 9:e54935).

After reviewing the raw image archive and figureassembly working files, we determined that the duplicated/similar regions were introduced during figure compilation rather than during the experiments. During early preparation of the multipanel layouts (while experiments were ongoing), we placed temporary representative micrographs into draft for internal review and saved as working files. Because these working files were stored in multiple folders and carried very similar filenames, some early composites were inadvertently reopened during final manuscript preparation and used as source material. When incorporated into the submission figures, we process them using routine formatting steps that include cropping to regions of interest, rescaling to fit panel dimensions, and (in two instances) 90° rotation to match panel orientation. Consequently, different crops from the same underlying micrograph produced either full duplication (after rotation) or partial overlap (when distinct regions of the same source image were used). No new image acquisition or experimental manipulation occurred at this stage. The errors reflect repeated selection of the same archived micrograph during assembly. The original raw images for each age/condition remain distinct and are archived separately.

Case 1 (WT testis panels): A single SAβGal micrograph originally used in Figure 1C (3monthold WT testis) was present in multiple intermediate working folders created during early figure drafting. During final assembly, it was (a) inserted correctly as Figure 1C, (b) inadvertently selected again while compiling Figure 1D (intended to show 6/9monthold WT testis) and then cropped/rescaled and rotated 90°, and (c) selected a third time while compiling Figure 5H (intended to show 6monthold WT testis), where a different cropped region of the same micrograph was used. Thus, one original raw image generated three derived panels through independent file selections and layout modifications.

Case 2 (tert/ testis panels): The SAβGal micrograph shown in Figure 1D (6/9monthold tert/ testis) was mistakenly reused in Figure 5H (intended to show 6monthold tert/ testis) after cropping/rescaling and 90° rotation. This reflects the same fileselection/processing error and therefore created reuse across different ages.

We have replaced the affected panels with nonduplicated images from the original experiments: Figure 1D now shows the correct 6/9monthold WT testis SAβGal panel, and Figure 5H now shows unique SAβGal panels for 6monthold WT and 6monthold tert/ testis. Each updated panel is traceable to a unique raw image file.

All quantitative analyses, statistical evaluations, and conclusions remain unchanged. We reexamined the entire article and did not identify any further errors. The authors agree with this correction.

The corrected Figure 1 (updated for panel D) is shown here in full:

**Figure 1:**
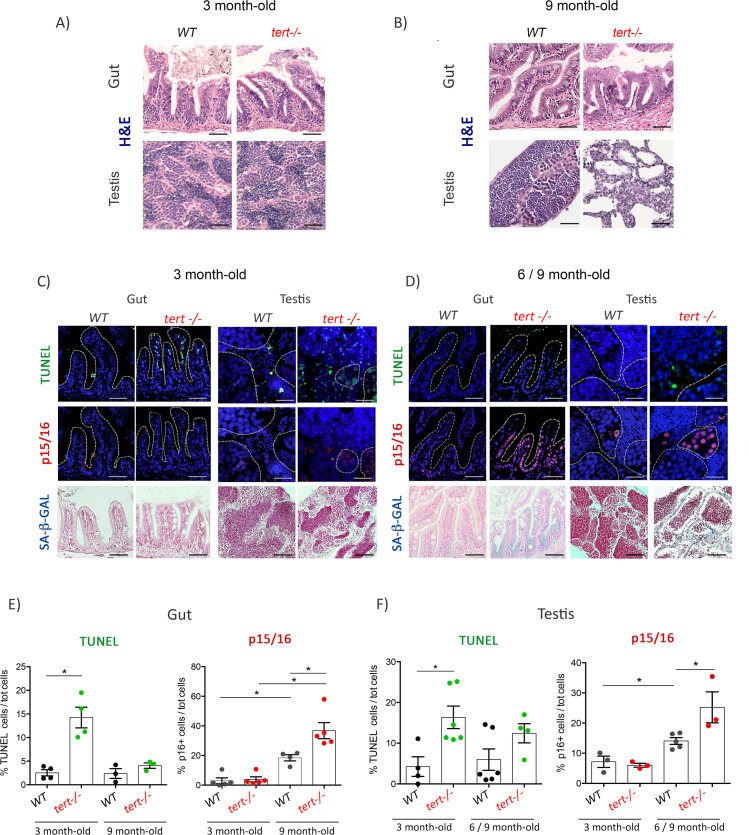


The originally published Figure 1 (showing the original panel D) is shown here in full for reference:

**Figure 2:**
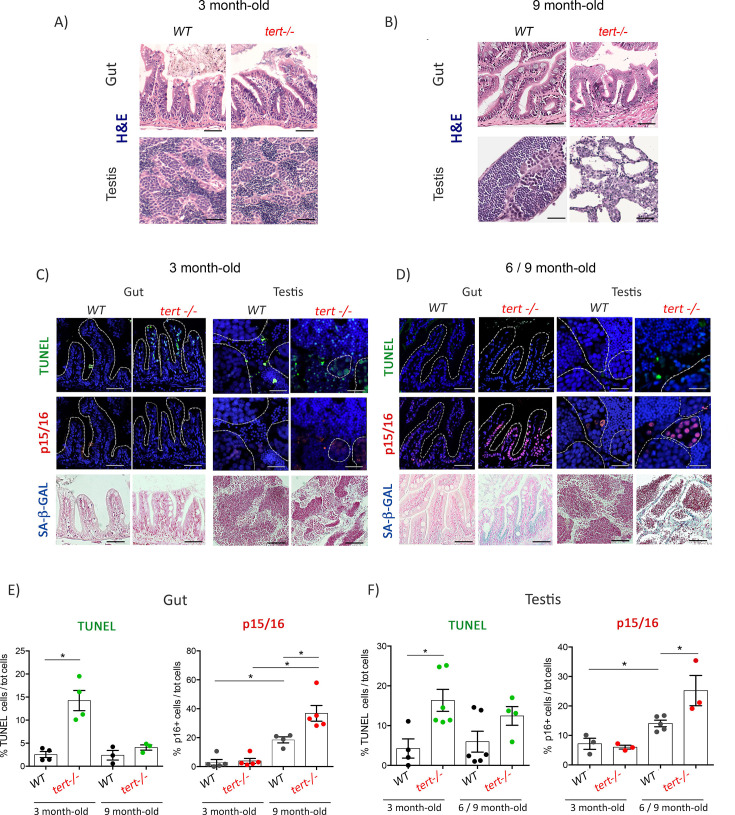


The corrected Figure 5 (updated for panel H) is shown here in full:

**Figure 3:**
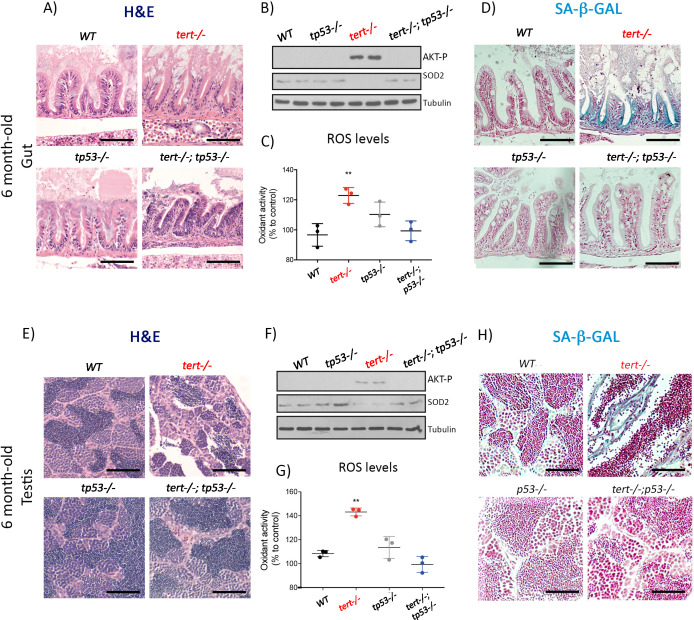


The originally published Figure 5 (showing the original panel H) is shown here in full for reference:

**Figure 4:**
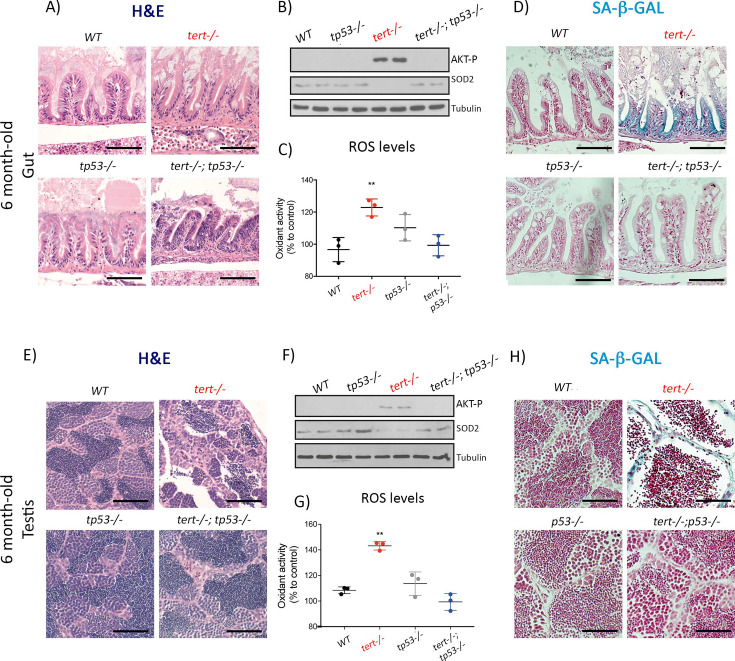


The article has been corrected accordingly.

