# Supplementary material for: Opposing p53 and mTOR/AKT promote an in vivo switch from apoptosis to senescence upon telomere shortening in zebrafish
Source: eLife. 2020 May 19;9:e54935. doi: 10.7554/eLife.54935 (PMC7237213; doi:10.7554/eLife.54935)
Supplement: Supplementary file 1. — Table listing the oligo-nucleotides used as primers for the RT-qPCR performed in this study. [file elife-54935-supp1.docx]

**Supplementary File 1 – List of primers used in RT-qPCR expression analysis.**

| Gene name | Primer sequences |
| --- | --- |
| *cdkn2a/b*  (p15/16) | forward – 5’ GAGGATGAACTGACCACAGCA  3’  reverse – 5’ CAAGAGCCAAAGGTGCGTTAC  3’ |
| *bcl2l1*  (Bcl-XL) | forward – 5’ GGGCTTGTTTGCTTGGTTGA 3’  reverse – 5’ AGAACACAGTGCACACCCTT 3’ |
| *cdkn1a*  (p21) | forward – 5’ CAGCGGGTTTACAGTTTCAGC 3’  reverse –5’ TGAACGTAGGATCCGCTTGT 3’ |
| *ppargc1a*  (PGC1a) | forward – 5’ CTGTGGAACCCCAGGTCTGAC3’  reverse – 5’ ACTCAGCCTGGGCCTTTTGCT 3’ |
| *rpl13a* | forward – 5’ TCTGGAGGACTGTAAGAGGTATG 3’   reverse – 5’ AGACGCACAATCTTGAGAGCAG  3’ |
